# Optic nerve sheath diameter increases during 12^◦^ head-down tilt in healthy volunteers: a crossover pilot study informing the design of a spaceflight countermeasure trial

**DOI:** 10.3389/fphys.2026.1890637

**Published:** 2026-07-28

**Authors:** Andrej Bergauer, Janez Urevc, Bianca Steuber, Karin Schmid–Zalaudek, Vid Pivec, Nandu Goswami

**Affiliations:** 1 Division of Physiology, Otto Loewi Research Center of Vascular Biology, Immunity and Inflammation, Medical University of Graz, Graz, Austria; 2 Department of Surgery, General Hospital (LKH Südweststeiermark), Wagna, Austria; 3 University of Ljubljana, Faculty of Mechanical Engineering, Ljubljana, Slovenia; 4 Center for Space and Aviation Health, Mohammed Bin Rashid University of Medicine and Health Sciences, Dubai, United Arab Emirates

**Keywords:** crossover design, head-down tilt, intracranial pressure, ONSD, optic nerve sheath diameter, SANS, spaceflight-associated neuro-ocular syndrome, transorbital ultrasound

## Abstract

**Background:**

Spaceflight-associated neuro-ocular syndrome (SANS) is hypothesized to arise from a headward fluid shift that elevates intracranial pressure and distends the optic nerve sheath. Head-down tilt (HDT) is the ground-based analog of microgravity, and optic nerve sheath diameter (ONSD) measured by transorbital B-mode ultrasound is a candidate non-invasive marker of intracranial pressure. Before a definitive trial in which lower body negative pressure (LBNP) is added to 12^◦^ HDT as a putative countermeasure, we conducted a pilot study to (i) confirm that 12^◦^ HDT detectably elevates ONSD with a high-frequency linear probe, (ii) identify the time of peak response, (iii) optimize the measurement schedule, and (iv) provide effect-size estimates for powering.

**Methods:**

Eight healthy young participants (4 women, 4 men; mean age 30.9 years) underwent two conditions in a randomized, balanced two-period crossover with a 14-day washout: supine (0^◦^ HDT) and 12^◦^ HDT. Bilateral transorbital B-mode ultrasound was recorded with a 17-MHz linear probe (Philips iU22) at 0, 30, 60, 90, 120, 150, 180, 210 and 240 min. Five frames per clip were measured offline and averaged across both eyes. The primary analysis was a 2-way repeated-measures ANOVA (Condition × Time) with Greenhouse–Geisser correction. Posthoc paired comparisons at each time point used Bonferroni adjustment. Gender was examined in a secondary mixed model.

**Results:**

The Condition × Time interaction was highly significant (*p*_GG_ *<* 0.001). ONSD did not differ between conditions up to 60 min but was larger under 12^◦^ HDT from 90 min onward, peaking at 150 min with a mean increment of 0.79 mm (95% CI 0.62–0.97), then partially regressing. No gender effect was detected, and baseline ONSD did not differ between visits, consistent with adequate washout.

**Conclusion:**

A 12^◦^ HDT exposure of ≥ 90 min produces a reproducible, large-magnitude ONSD increase peaking at 150 min in healthy adults; a 0–180 min measurement window is sufficient. These data support a full three-arm crossover trial of LBNP as a candidate countermeasure, with graduated lower-limb compression included as a mechanistic positive control to verify that headward fluid redistribution is the aggravating factor.

## 1 Introduction

Long-duration exposure to microgravity has been consistently associated with physiological deconditioning that affects most organ systems (Demontis et al., 2017; Goswami et al., 2026, 2021). Among these adaptations, microgravity has been reported to produce structural and functional ocular changes including optic disc edema, posterior globe flattening, choroidal folds and a hyperopic shift, collectively termed spaceflight-associated neuro-ocular syndrome (SANS) (Stenger et al., 2019; Paez et al., 2020; Macias et al., 2020). The leading pathophysiological hypothesis attributes SANS to a headward fluid shift that elevates central venous pressure, raises intracranial pressure and distends the optic nerve sheath, with secondary remodeling of the lamina cribrosa and posterior sclera (Mader et al., 2021; Rohr et al., 2020; Wostyn and De Deyn, 2022; Harris et al., 2022). Persistent globe flattening years after return from the International Space Station (Mader et al., 2021) and quantitative MRI evidence of optic nerve and sheath remodeling (Rohr et al., 2020) support a chronic, structural component of the syndrome.

Optic nerve sheath diameter (ONSD) measured by transorbital B-mode ultrasound has been validated against invasive intracranial pressure (ICP) monitoring and against quantitative MRI in a range of clinical contexts, including traumatic brain injury, idiopathic intracranial hypertension, and acute mountain sickness (Geeraerts et al., 2008; Fagenholz et al., 2009; Rajajee et al., 2011; Liu et al., 2017; Robba et al., 2018; Dubourg et al., 2011). Meta-analyses consistently report an ONSD threshold of approximately 5.0–5.9 mm for the detection of elevated ICP, with summary diagnostic odds ratios above 50 (Robba et al., 2018; Dubourg et al., 2011). The technique is non-invasive, compact and portable, and is therefore an attractive candidate biomarker for long-duration spaceflight and for terrestrial analog studies.

Marshall-Goebel and colleagues established 12^◦^ HDT as the closest ground-based analog of microgravity capable of reproducing the SANS phenotype within hours of exposure, and showed that lower body negative pressure (LBNP), a well-characterized experimental tool for redistributing blood volume from the thorax toward the lower extremities (Goswami et al., 2019a; Goswami, 2023), can attenuate the ONSD response (Marshall-Goebel et al., 2017b; Marshall-Goebel et al., 2017a). Although these findings establish 12^◦^ HDT as a usable analog and LBNP as a plausible countermeasure, the temporal dynamics of the ONSD response, the minimum exposure needed to elicit it, and the effect size to expect have not been characterized in a controlled crossover; these gaps directly determine how a definitive countermeasure trial must be designed and powered. We therefore framed the present pilot around three methodological questions: (i) the time at which the ONSD response to HDT plateaus or peaks; (ii) the minimum exposure duration that still yields a discernible effect; and (iii) the realistic effect size that should drive a sample-size calculation. The crossover design also imposes its own validity assumptions: an adequate inter-period washout and an absence of carryover.

Beyond establishing feasibility, the present study was designed to provide the methodological foundation for a definitive countermeasure trial. Specifically, it aimed to characterize the temporal dynamics of the ONSD response, quantify its variability, and generate effect-size estimates required for prospective study design.

We therefore conducted a randomized, balanced two-period crossover pilot study in eight healthy young participants, in which ONSD was tracked every 30 min for 4 h during both supine rest and 12^◦^ HDT. Our aim was to confirm a measurable ONSD increase under 12^◦^ HDT with a 17-MHz linear probe and to characterize its temporal profile. We further intended to derive effect-size and variance estimates for a definitive three-arm trial in which LBNP will be evaluated as a candidate countermeasure designed to reduce the headward fluid shifts, while graduated lower-limb compression will be included as a mechanistic positive control expected to produce the opposite effect by augmenting the headward redistribution of blood volume. Finally, we sought to verify the validity of the crossover design itself, by assessing period and carryover effects.

## 2 Materials and methods

### 2.1 Participants

Eight healthy adult participants (4 women, 4 men) were enrolled; demographic and baseline characteristics are summarized in **Table 1**. Exclusion criteria were any known ophthalmologic disease, prior orbital trauma or surgery, known intracranial pathology, and any general medical condition contraindicating prolonged head-down posture. The study protocol was reviewed by the Medical Ethics Committee of the University Medical Centre Maribor (UKC-MB), which, in view of the non-invasive nature and limited scope of the pilot study, issued an exempt determination and waived the requirement for formal ethics review (UKC-MB-KME-X/20, 15 January 2020). All participants provided written formed consent.

**Table 1:** Demographic and baseline characteristics of the eight participants. Sequence “AB” indicates that Period 1 was 0^◦^ HDT and Period 2 was 12^◦^ HDT; “BA” indicates the reverse.

Subject	Sex	Age (y)	Sequence	Baseline ONSD,	Baseline ONSD,
				$0^{\circ }$ **HDT (mm)**	$12^{\circ }$ **HDT (mm)**
1	M	23	BA	4.51	4.59
2	F	25	AB	4.48	4.45
3	M	38	BA	5.36	5.38
4	F	36	AB	4.83	4.91
5	M	34	AB	5.61	5.58
6	F	28	BA	4.42	4.38
7	M	24	AB	4.63	4.59
8	F	39	BA	4.71	4.78
**Women** ( n=4)		32.0±6.9	2 AB/2 BA	4.61±0.20	4.63±0.25
**Men** ( n=4)		29.8±7.5	2 AB/2 BA	5.03±0.55	5.03±0.53
**All** ( n=8)		30.9±6.8	4 AB/4 BA	4.82±0.44	4.83±0.44

Baseline ONSD is the value measured at *t* = 0 min for each condition. Summary rows show mean ± standard deviation; SD is omitted for *n* = 8 where age range is given in the body text.

Bold entries in the last three rows are group summary statistics (mean ± standard deviation) for the Women (n = 4), Men (n = 4), and All (n = 8) subgroups. Bold column headers indicate table field names.

### 2.2 Study design

The study used a randomized, balanced two-period two-treatment crossover design (**Table 2**). The two test conditions were supine (0^◦^ HDT, condition A) and 12^◦^ head-down tilt (condition B). The order (AB or BA) was randomized by Latin square. A washout interval of ≥ 14 days separated the two periods to minimize carryover.

**Table 2:** Latin-square allocation of the two conditions across the two periods.

	Subject							
Period	1	2	3	4	5	6	7	8
1	B	A	B	A	A	B	A	B
2	A	B	A	B	B	A	B	A

Condition A = 0^◦^ head-down tilt (supine); Condition B = 12^◦^ head-down tilt. Subjects 1–8 were assigned at random to the sequence in the table.

### 2.3 Ultrasound protocol

Transorbital B-mode ultrasound was performed with a Philips iU22 system (Philips Healthcare, Bothell, WA, USA) using a 17-MHz high-frequency linear probe. Participants kept their eyes closed and looked in the primary position. The probe was placed transversely over the closed upper eyelid through coupling gel. Short cine clips of 5–10 s (median 268 frames) were recorded for each eye at *t* ∈{0,30,60,90,120,150,180,210,240} min relative to the onset of the test condition.

### 2.4 Measurements

Clips were analyzed offline with Horos v.3.3.6 (Horosproject.org, sponsored by Nimble Co LLC d/b/a Purview, Annapolis, MD, USA) by a single trained rater. The optic nerve sheath diameter was measured 3 mm posterior to the globe, perpendicular to the optic nerve axis, between the outer borders of the hypoechoic optic nerve sheath. Five technically optimal, non-consecutive frames were selected per clip; five repeat measurements were taken on each frame for each eye and averaged. The left- and right-eye averages were further averaged to yield a single ONSD value per participant per time point per condition.

### 2.5 Statistical analysis

The pre-specified primary analysis was a two-way repeated-measures ANOVA. Condition (0^◦^, 12^◦^) and Time (9 levels) were the within-subject factors, with Subject as the random error term. Sphericity was assessed by Mauchly’s test (Greenhouse and Geisser, 1959), and the Greenhouse–Geisser correction was applied where sphericity was violated. Normality of the residuals was checked by Shapiro–Wilk test and Q–Q plots. Effect sizes are reported as generalized eta squared ( $\eta_{G}^{2}$^)^.

For the time-resolved *post-hoc* comparison of conditions, paired *t*-tests were performed at each of the 9 time points with Bonferroni adjustment (9 comparisons). Effect sizes for the *post-hoc* comparisons are reported as Cohen’s *d_z_*for paired samples.

A secondary mixed ANOVA added Gender as a between-subject factor. Because each cell contained only four participants, this analysis was treated as exploratory.

To validate the crossover design we tested for a period effect on baseline ONSD (*t* = 0 min) using a paired *t*-test comparing each participant’s first-visit baseline with their second-visit baseline (Hills and Armitage, 1979). The 14-day washout was deemed adequate if no significant period effect was detected.

All analyses were performed in Python (v.3.10) using pandas, statsmodels, pingouin and SciPy. The SPSS syntax and a fully reproducible Python script are available from the corresponding author on reasonable request. Two-sided *α* = 0.05 throughout.

## 3 Results

### 3.1 Descriptive statistics

The complete dataset comprised 144 ONSD observations (8 participants × 2 conditions × 9 time points), with no missing values. Group means and standard deviations are reported in **Table 3**. The mean (± SD) baseline ONSD at *t* = 0 min was 4.82 ± 0.44 mm under 0^◦^ HDT and 4.83 ± 0.44 mm under 12^◦^ HDT, with no detectable between-condition difference at baseline (paired *t* = 0.70, *p* = 0.51).

**Table 3:** Descriptive statistics of optic nerve sheath diameter (mm) by condition and time point in eight participants.

Time (min)	0^◦^ HDT	12^◦^ HDT
0	4.82 ± 0.44	4.83 ± 0.44
30	5.29 ± 0.31	5.17 ± 0.51
60	5.42 ± 0.41	5.38 ± 0.47
90	5.31 ± 0.37	5.50 ± 0.41
120	5.23 ± 0.36	5.66 ± 0.40
150	5.18 ± 0.39	5.97 ± 0.48
180	5.03 ± 0.41	5.72 ± 0.36
210	5.08 ± 0.41	5.52 ± 0.37
240	5.02 ± 0.42	5.39 ± 0.41

Values are mean ± standard deviation.

### 3.2 Primary analysis: time *×* condition interaction

**Figure 1:**
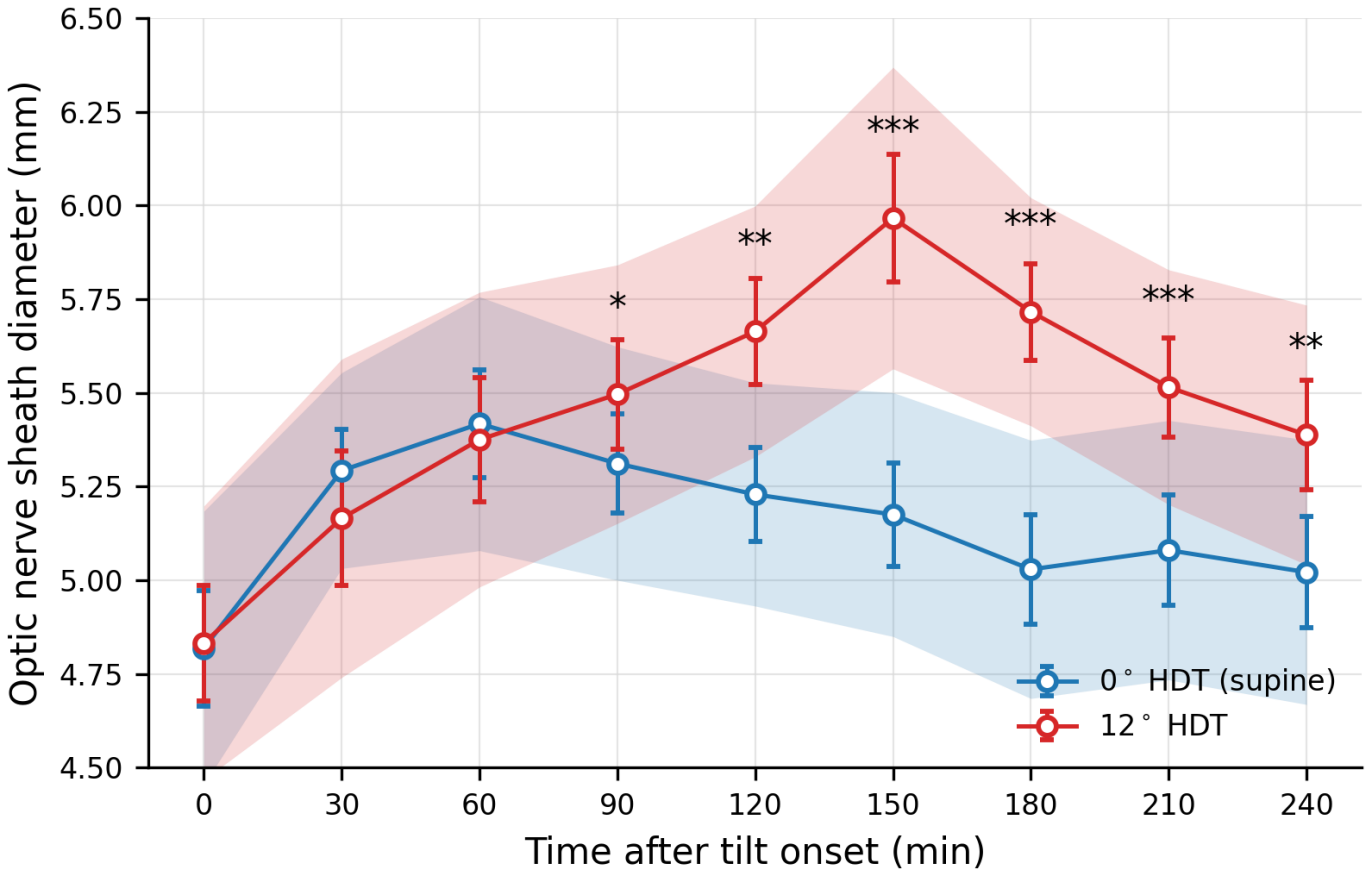
Group mean time course of optic nerve sheath diameter (ONSD) under 0^◦^ and 12^◦^ head-down tilt. Symbols, mean (*n* = 8); error bars, standard error of the mean; shaded ribbon, 95% confidence interval. Asterisks denote Bonferroni-adjusted significance of the paired 12^◦^ vs. 0^◦^ comparison at each time point (^∗^*p <* 0.05; ^∗∗^*p <* 0.01; ^∗∗∗^*p <* 0.001).

**Figure 2:**
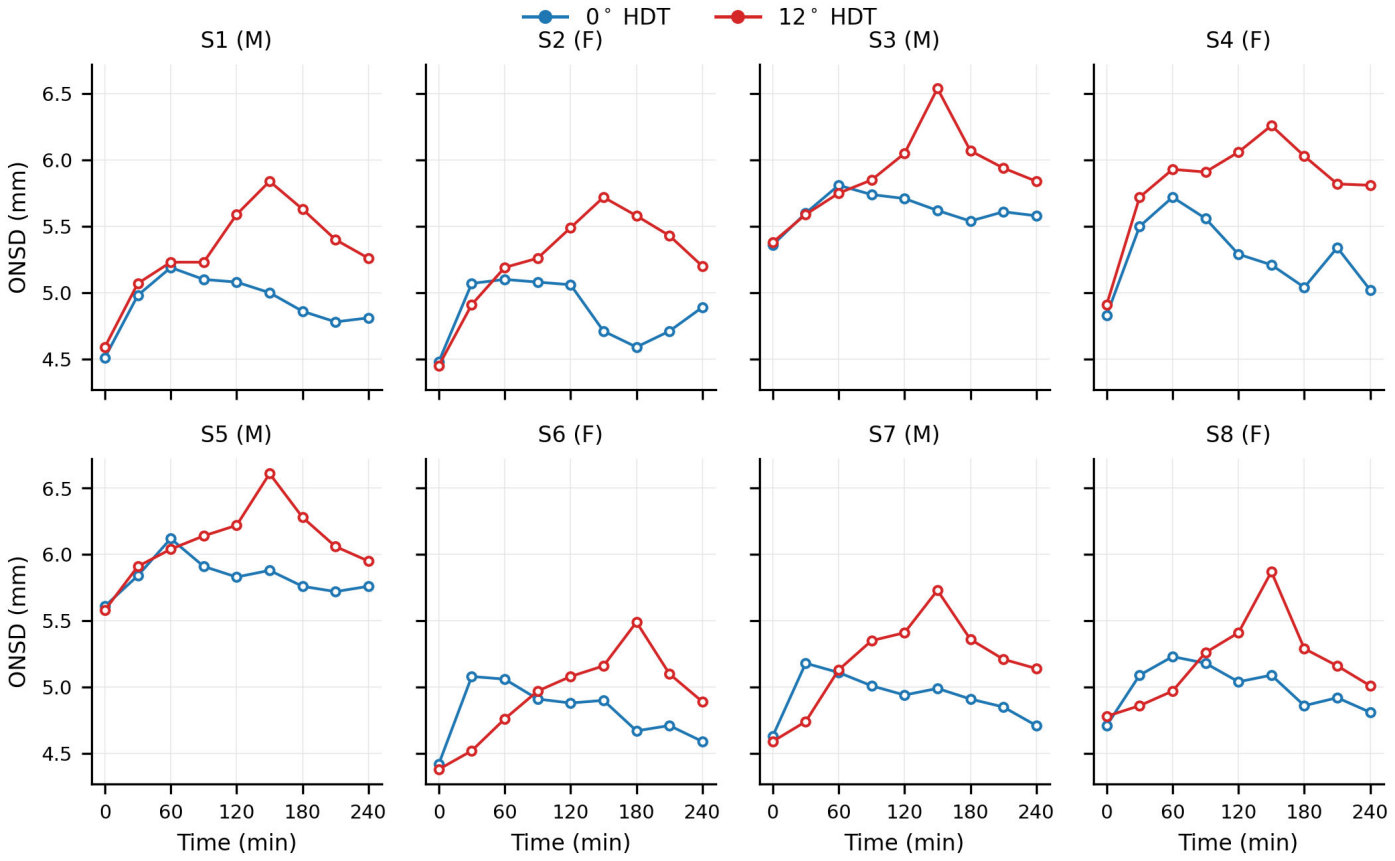
Individual ONSD trajectories for the eight participants under both conditions. Each panel shows one participant; F, female; M, male. Blue, 0^◦^ HDT; red, 12^◦^ HDT.

The two-way repeated-measures ANOVA revealed significant main effects of Condition (*F*(1,7) = 41.24, *p <* 0.001, $\eta_{G}^{2}$ = 0.13) and Time (*F*(8,56) = 59.62, *p <* 0.001, $\eta_{G}^{2}$ = 0.21), and a highly significant Condition × Time interaction (*F*(8,56) = 40.45, *p <* 0.001, $\eta_{G}^{2}\;$= 0.13; **Table 4**). Mauchly’s test indicated a violation of sphericity for Time under the 0^◦^ HDT condition (*χ*^2^ = 148.2, *p <* 0.001, *ϵ* = 0.32); after Greenhouse–Geisser correction the interaction remained significant (*p*_GG_ *<* 0.001). **Figure 1** shows the group mean time course for both conditions; **Figure 2** shows the individual trajectories.

**Table 4:** Two-way repeated-measures ANOVA results (Condition × Time) for ONSD in eight participants.

Effect	F	$df_{1}$	$df_{2}$	p	$p_{\text{GG}}$	$\eta_{G}^{2}$
Condition	41.24	1	7	<0.001	<0.001	0.13
Time	59.62	8	56	<0.001	<0.001	0.21
Condition × Time	40.45	8	56	<0.001	<0.001	0.13

$\eta_{G}^{2}$, generalized eta-squared; $p_{\text{GG}}$, Greenhouse–Geisser corrected *p*-value.

### 3.3 *Post-hoc* time-resolved comparison

**Figure 3:**
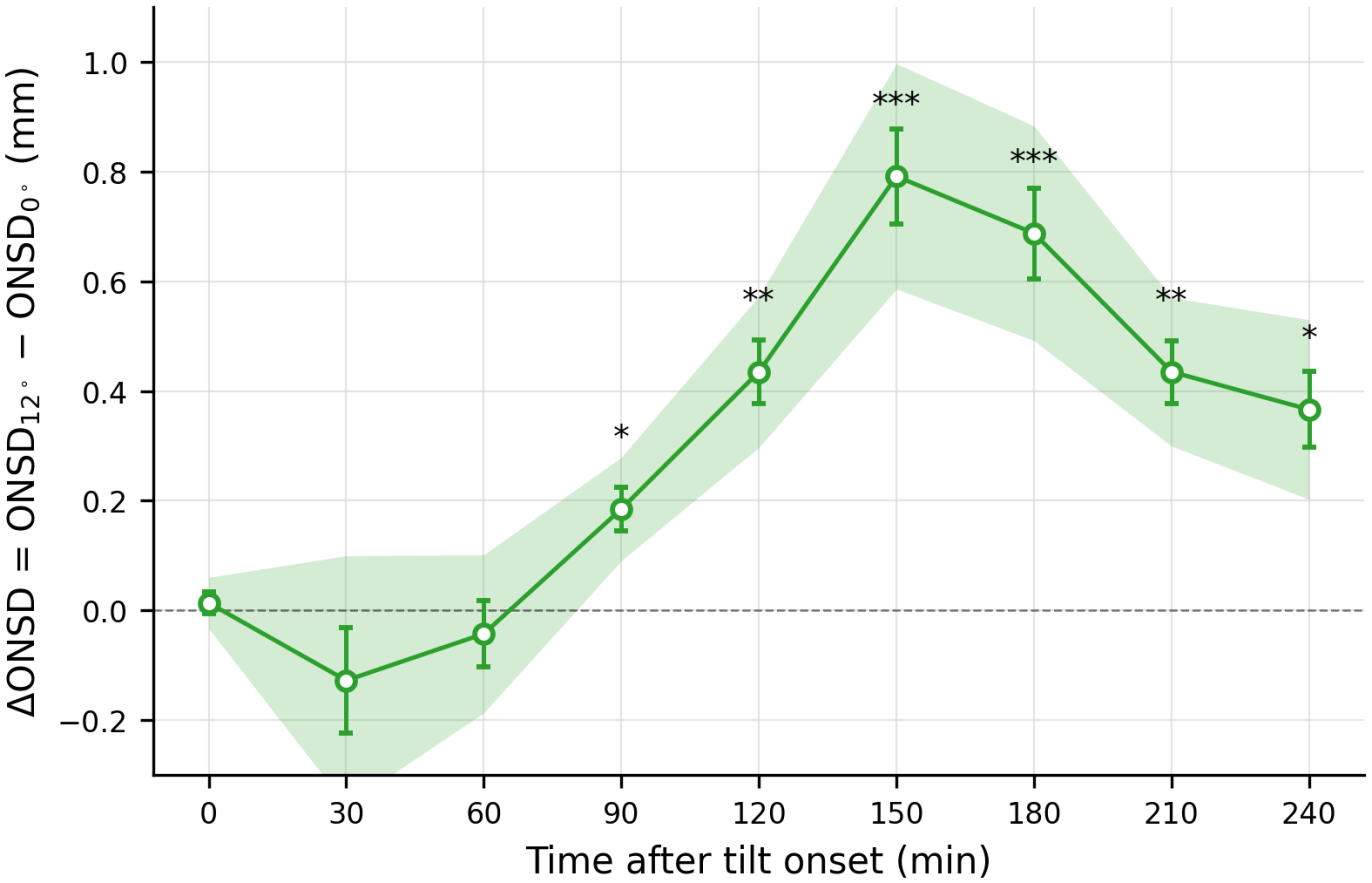
Time course of the mean within-subject difference ΔONSD = ONSD_12_^◦^ −ONSD_0_^◦^. Symbols, mean (*n* = 8); error bars, standard error; shaded ribbon, 95% confidence interval. Significance markers as in **Figure 1**.

Bonferroni-adjusted paired comparisons at each time point (**Table 5**; **Figure 3**) showed that ONSD did not differ between conditions at *t* ∈{0,30,60} min (*p*_Bonf_ = 1.00). From *t* = 90 min onward, ONSD under 12^◦^ HDT was significantly larger than under 0^◦^ HDT, and the difference grew monotonically up to *t* = 150 min, where it reached 0.79 mm (95% CI 0.62–0.97; Cohen’s *d_z_*= 3.22; *p*_Bonf_ *<* 0.001). The difference declined after 150 min but remained statistically significant up to the last time point at 240 min (Δ = 0.37 mm; *p*_Bonf_ = 0.010).

**Table 5:** *Post-hoc* paired comparison of 12^◦^ HDT vs. 0^◦^ HDT at each time point.

Time (min)	Δ (mm)	*t*(7)	*p* (raw)	*p*Bonf	*d_z_*
0	+0.014	+0.70	0.506	1.000	0.25
30	−0.128	−1.33	0.226	1.000	−0.47
60	−0.043	−0.70	0.507	1.000	−0.25
90	+0.185	+4.65	0.002	0.021	1.64
120	+0.435	+7.48	*<* 0.001	0.001	2.64
150	+0.791	+9.11	*<* 0.001	*<* 0.001	3.22
180	+0.688	+8.31	*<* 0.001	*<* 0.001	2.94
210	+0.435	+7.61	*<* 0.001	0.001	2.69
240	+0.366	+5.29	0.001	0.010	1.87

Δ, mean (12^◦^ – 0^◦^); *d_z_*, Cohen’s *d* for paired samples; *p*_Bonf_, *p*-value after Bonferroni adjustment for 9 comparisons.

### 3.4 Secondary analysis: gender

**Figure 4:**
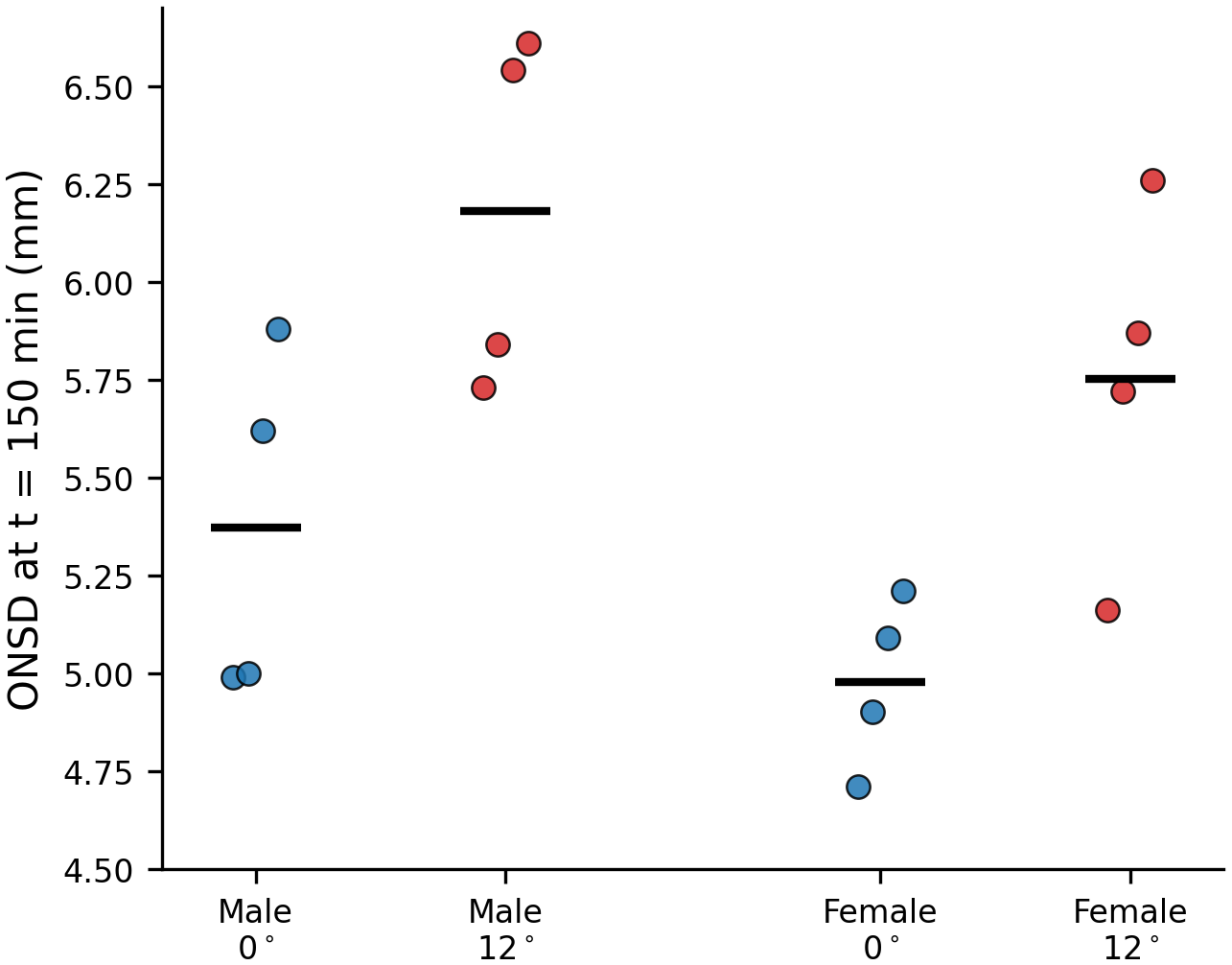
Individual ONSD values at the peak time point (*t* = 150 min) by condition and gender. Each dot is one participant (*n* = 4 per cell); horizontal black bars indicate the cell mean. Blue, 0^◦^ HDT; red, 12^◦^ HDT. No significant gender main effect or gender × condition interaction was detected; the analysis is exploratory only.

The exploratory mixed ANOVA detected no main effect of Gender ( $F(1,\;6)=1.53,\;p=0.26,\;\eta_{G}^{2}=0.20$) and no significant interaction of Gender with Time, Condition or their interaction (all *p >* 0.13). With only four participants per cell, however, the study was not powered to detect a plausibly sized gender effect; this analysis should be interpreted as hypothesis-generating only. **Figure 4** shows the gender-stratified ONSD distribution at the peak time point.

### 3.5 Validation of the crossover design

Baseline (*t* = 0 min) ONSD did not differ between the first and second visit (mean Period 2 – Period 1 = −0.02 mm; paired *t* = −0.99, *p* = 0.36). This is consistent with an adequate 14-day washout and no detectable carryover between conditions.

## 4 Discussion

In this pilot crossover study of healthy young adults, 12^◦^ HDT produced a clear, time-resolved increase in transorbitally measured ONSD relative to supine rest. The Condition × Time interaction was the dominant statistical signal (*F*(8,56) = 40.4, *p <* 0.001): under HDT, ONSD rose from a baseline of 4.83 mm to a peak of 5.97 mm at 150 min, whereas under supine rest the post-baseline rise was modest and transient, peaking near 60 min at 5.42 mm and declining toward baseline thereafter. The maximum between-condition difference of 0.79 mm at *t* = 150 min is of the order reported by Marshall-Goebel et al. (Marshall-Goebel et al., 2017b) for the same tilt angle and exceeds the threshold widely used clinically to flag elevated ICP (Robba et al., 2018; Dubourg et al., 2011).

Our analysis takes two methodological positions worth spelling out. We treated Condition and Time as within-subject factors in a 2-way repeated-measures model and kept Gender out of the primary analysis. A mixed model with Gender as a between-subject factor was run in parallel, but with only four participants per cell that approach has too little power to detect plausible gender effects and dilutes the test of the primary contrast; in our hands it confirmed the qualitative conclusions of the within-subject model without contributing any meaningful information about sex differences. We also avoided reducing each participant’s response to a single “maximum minus baseline” contrast. Because the timing of the maximum varies between participants, a *post-hoc* selection of that peak inflates Cohen’s *d* and yields unrealistic power projections. Reporting Bonferroni-adjusted simple effects at every time point answers a more clinically relevant question, namely *when* the ONSD signal emerges, and the pre-specified comparison at *t* = 150 min (*d_z_*= 3.22) provides an honest effect-size estimate for sample-size planning.

The temporal profile we observed has direct implications for the main-study protocol. ONSD did not differ between conditions before 90 min and peaked at 150 min, so an exposure of 150–180 min appears sufficient to capture the maximum response. Extending the protocol to 240 min, as in this pilot, adds little information because the signal has begun to attenuate. The originally envisioned 5-hour exposure can therefore be safely shortened.

The pilot also informs the sample-size planning of the main three-arm crossover trial. For the primary Condition × Time contrast the pilot effect size is large (*η_G_*^2^ = 0.13; Cohen’s *d_z_*= 3.22 at the peak), so detection of the average HDT response will be trivial with any realistic sample. The more demanding requirement is detection of a Sex × Condition interaction, which the present data cannot quantify because the secondary gender comparison had only four participants per cell; nonetheless, empirical evidence of sex-specific responses to graded central hypovolemia under LBNP (Goswami et al., 2019b, 2020) motivates the prospective sex stratification of the main study. The effect size relevant for planning is therefore the smallest sex difference in the HDT-induced ONSD increment that is judged clinically meaningful. The pilot within-subject standard deviation of the (HDT12 − HDT0) difference was approximately 0.25 mm. Taking a between-sex difference of 0.30 mm in the HDT response (about 40% of the average increment) as the smallest effect of interest, this corresponds to roughly 11 participants per sex (22 total) at *α* = 0.05 (twosided) and 80% power. A more conservative smallest effect of interest of 0.20 mm raises the requirement to approximately 24 per sex (48 total). A 10–15% allowance for non-completers gives a recommended enrolment of 24–56 participants with balanced sex allocation. The LBNP arm is expected to *attenuate* the HDT-induced ONSD increase, whereas the graduated lower-limb compression arm (included as a mechanistic positive control) is expected to produce the *opposite* effect, augmenting the HDT-induced increase by adding to the headward redistribution of blood volume. Both arms therefore have anticipated effect sizes at least as large as the HDT-only response and do not increase the sample-size requirement.

The absence of a period effect on baseline ONSD (Period 2 – Period 1 = −0.02 mm; *p* = 0.36) and the balanced Latin-square allocation support the validity of the crossover design and indicate that a 14-day washout is adequate, which is consistent with the design requirements of the main study, in which crossover between three conditions (control HDT; HDT + LBNP as the candidate countermeasure; HDT + graduated lower-limb compression as a mechanistic positive control) will be necessary.

### 4.1 Limitations

Several limitations should be acknowledged together with the way each is addressed by the present design or by the planned main study.

First, the sample size of eight is small. This is by design: the study was conceived and powered as a pilot, not as a confirmatory test of a population-level hypothesis. A formal *a priori* sample-size calculation was therefore not performed; instead, the pilot was sized to characterize the magnitude and temporal profile of the HDT-induced ONSD response and to estimate the within-subject variance needed for prospective powering. The sample-size projection presented in the preceding paragraph (24–56 participants with balanced sex allocation) follows directly from the variance observed here and provides the basis for the main study’s *a priori* power calculation.

Second, all measurements were performed by a single rater. While this maximizes within-study consistency and removes inter-rater variability as a confound for the pilot, inter-rater reliability has not been formally quantified. The main study will use at least two independent raters and will report intraclass correlation coefficients on a representative subset of clips.

Third, the 17-MHz linear probe has excellent axial resolution but limited depth penetration. In the eight pilot participants the orbit was successfully imaged in every recording, so no participant was excluded for image-quality reasons; nonetheless, deeper orbits may pose difficulty in a larger sample. A lower-frequency backup probe will be available in the main study as a per-protocol substitution where image quality is judged inadequate.

Fourth, bilateral averaging assumes inter-eye symmetry, which holds in healthy participants but may not in asymmetric pathology. Because the present cohort is healthy and asymptomatic, this assumption is reasonable here; the main study will additionally report left- and right-eye values as a secondary outcome to detect inter-ocular asymmetry, should it arise.

Fifth, the precise time at which ONSD peaks may shift when LBNP or the compression positive control is added. For exactly this reason the main study will retain the dense 0–180 min measurement grid validated in this pilot rather than collapsing the response to a single time point, allowing the temporal profile to be re-characterized for each intervention arm.

Sixth, this pilot used a 12^◦^ head-down tilt, whereas the majority of ground-based microgravity analog studies use the more established 6◦ angle developed for long-duration head-down bed-rest protocols. The 12◦ angle was chosen because it is a well-established acute ground-based analog for eliciting the SANS-related headward fluid shift, following the paradigm established by Marshall-Goebel et al (Marshall-Goebel et al., 2017b; Marshall-Goebel et al., 2017a). This limits the direct quantitative comparability of our absolute ONSD values with the wider 6^◦^ HDT literature. Nevertheless, the chosen protocol aligns the study with the smaller body of work specifically targeting the acute SANS phenotype.

## 5 Conclusion

A 12^◦^ HDT exposure of 90 min or more produces a reproducible, large-magnitude increase in optic nerve sheath diameter measurable by 17-MHz transorbital B-mode ultrasound in healthy young adults, with a peak at 150 min and partial regression thereafter. A protocol with 0–180 min of exposure and ONSD measurement every 30 min is sufficient to capture the response. These results support the design of a randomized three-arm crossover trial in which LBNP is evaluated as a candidate countermeasure for the SANS-related ONSD increase, and graduated lower-limb compression is included as a mechanistic positive control to verify that headward fluid redistribution is the aggravating factor. Adequate inter-period washout is confirmed at 14 days.

## Data Availability

The original contributions presented in the study are included in the article/**Supplementary Material**. Further inquiries can be directed to the corresponding author.

## Supplementary material

The complete de-identified dataset is provided as a Supplementary Table (**Supplementary Table 1**): optic nerve sheath diameter measurements for each participant at each time point under 0° and 12° head-down tilt (72 rows; 8 participants × 9 time points × 2 conditions = 144 observations; no missing values).
