# Supplementary material for: Optic nerve sheath diameter increases during 12◦ head-down tilt in healthy volunteers: a crossover pilot study informing the design of a spaceflight countermeasure trial
Source: Front Physiol. 2026 Jul 28;17:1890637. doi: 10.3389/fphys.2026.1890637 (PMC13457123; doi:10.3389/fphys.2026.1890637)
Supplement: Supplementary file 1 [file DataSheet1.pdf]

Supplementary Material

1 SUPPLEMENTARY TABLE

Table S1: Complete de-identified dataset of optic nerve sheath diameter (ONSD) measurements from the head-down tilt (HDT) pilot study. Values are ONSD in millimetres for each participant at each time point under 0° (supine) and 12° head-down tilt. F, female; M, male. The dataset comprises 8 participants × 9 time points × 2 conditions = 144 observations, with no missing values.

Subject	Sex	Time (min)	ONSD 0° (mm)	ONSD 12° (mm)
1	M	0	4.51	4.59
1	M	30	4.98	5.07
1	M	60	5.19	5.23
1	M	90	5.10	5.23
1	M	120	5.08	5.59
1	M	150	5.00	5.84
1	M	180	4.86	5.63
1	M	210	4.78	5.40
1	M	240	4.81	5.26
2	F	0	4.48	4.45
2	F	30	5.07	4.91
2	F	60	5.10	5.19
2	F	90	5.08	5.26
2	F	120	5.06	5.49
2	F	150	4.71	5.72
2	F	180	4.59	5.58
2	F	210	4.71	5.43
2	F	240	4.89	5.20
3	M	0	5.36	5.38
3	M	30	5.60	5.59
3	M	60	5.81	5.75
3	M	90	5.74	5.85
3	M	120	5.71	6.05
3	M	150	5.62	6.54
3	M	180	5.54	6.07
3	M	210	5.61	5.94
3	M	240	5.58	5.84
4	F	0	4.83	4.91
4	F	30	5.50	5.72
4	F	60	5.72	5.93
4	F	90	5.56	5.91
4	F	120	5.29	6.06
4	F	150	5.21	6.26
4	F	180	5.04	6.03
4	F	210	5.34	5.82
4	F	240	5.02	5.81
5	M	0	5.61	5.58
5	M	30	5.84	5.91
5	M	60	6.12	6.04
5	M	90	5.91	6.14
5	M	120	5.83	6.22

continued on next page

Supplementary Table S1 – continued from previous page

Subject	Sex	Time (min)	ONSD 0° (mm)	ONSD 12° (mm)
5	M	150	5.88	6.61
5	M	180	5.76	6.28
5	M	210	5.72	6.06
5	M	240	5.76	5.95
6	F	0	4.42	4.38
6	F	30	5.08	4.52
6	F	60	5.06	4.76
6	F	90	4.91	4.97
6	F	120	4.88	5.08
6	F	150	4.90	5.16
6	F	180	4.67	5.49
6	F	210	4.71	5.10
6	F	240	4.59	4.89
7	M	0	4.63	4.59
7	M	30	5.18	4.74
7	M	60	5.11	5.13
7	M	90	5.01	5.35
7	M	120	4.94	5.41
7	M	150	4.99	5.73
7	M	180	4.91	5.36
7	M	210	4.85	5.21
7	M	240	4.71	5.14
8	F	0	4.71	4.78
8	F	30	5.09	4.86
8	F	60	5.23	4.97
8	F	90	5.18	5.26
8	F	120	5.04	5.41
8	F	150	5.09	5.87
8	F	180	4.86	5.29
8	F	210	4.92	5.16
8	F	240	4.81	5.01
